# Challenges constraining sustainable household food security in Kasarani sub-county, Nairobi County, Kenya

**DOI:** 10.11604/pamj.2024.49.126.28779

**Published:** 2024-12-18

**Authors:** Namenge Philip Tendet, George Alliwa Makalliwa, Daniel Nyamongo Sagwe

**Affiliations:** 1 Department of Public Health, College of Health Services, Jomo Kenyatta University of Science and Technology, Nairobi, Kenya

**Keywords:** Food security, social-economic, urban farmers

## Abstract

**Introduction:**

as cities continue to grow rapidly due to urbanization, urban dwellers have experienced food insecurity resulting from an upsurge in the cost of staple foods. The study investigated the challenges constraining sustainable household food security in Kasarani sub-county, Nairobi County, Kenya.

**Methods:**

by the use of a descriptive survey design, questionnaires and expert guidance, data was collected for analysis using Statistical Package for Social Sciences (SPSS). The internal consistency and reliability coefficients of 0.731 and 0.881 were used to test reliability and consistency of the research instruments.

**Results:**

from a sample of 328 farmers, 65.55% (n=215) were males while 34.45% (n=113) were female. The married, divorced, single and widowed were 63.41 (n=208), 23.78 (n=78), 5.79 (19) and 7.01% (n=23), respectively. Casual laborers, business people and formally employed ones made up 41.46 (n=136), 33.54 (110) and 25% (n=82), respectively. 32.93% (n=108) practiced urban farming while 24.70% (n=81) were involved in commercial purposes and food security. 10.98% (n=36) practiced urban farming for income diversification while 12.19% (n=40) did it as a hobby/custom and 2.13% (n=7) were unspecified. 63.11% (n=207) earned between 40-80 USD. 36.89% (n=121) earned between 80-120 USD while 4.02% (n=13) earned more than 120 USD. Urban farming was constrained by flooding (MN-3.66) followed by lack of rain (MN-3.52) pest/diseases (MN=3.49), poor yields (MN=3.28) and soil erosion (MN=3.23).

**Conclusion:**

the study recommends adherence to weather forecasting to reduce on the effects of flooding, adoption of measures to prevent soil erosion and usage of pesticides whenever applicable. A policy and institutional framework for the sector need to be established to enable urban farmers to mutually benefit from urban farming.

## Introduction

More than 50% of the world´s population lives in cities. It is expected that by 2020 and 2035, urban dwellers in Asia and Africa respectively will make up half of the population of the two regions [1]. The number of highly vulnerable urban residents living in urban informal settlements worldwide currently stands at over 900 million people. Most of these people are found in low and middle-income countries [2]. Demographic estimates indicate that by the year 2000, 37% of the African population was expected to be living in urban areas while in 2025, the urban population is expected to have grown to more than 50% [2]. Even though the estimates are lesser than in other continents, the annual population growth rates in the urban areas are highest in Africa [3]. The global number of hungry people keeps rising with the years and stands at one in three people in sub-Saharan Africa, which has a population of approximately 183 million people [2]. The fast population increase in urban areas has alerted concerns about food security in terms of its accessibility and availability [4]. The end result is an effect on public health as people end up malnourished. Close to a million more Kenyans have in the past two decades joined the ranks of those who cannot afford a decent meal, school fees, and adequate health care [5]. This sums the number of people who are living below the poverty line of 1 US Dollar daily to 12 million [4]. Of these, 15 % live in rural areas while the rest are found in urban areas. As a result of the escalating trends in urban poverty, many households, especially those in the low-income bracket, suffer from food insecurity and malnourishment. The foregoing discussions indicate a high prevalence of hunger and malnourishment and urban household food security in Kenya. The definition of food security thus evolves and shifts from simple food availability to incorporating issues of food adequacy, supplies, and secure access at the individual, household, national, and international levels [6]. The broad domain of this research is food security which implicates public health for urban dwellers. The study aimed to investigate challenges constraining sustainable household food security in Kasarani sub-county, Nairobi County, Kenya.

## Methods

**Study design and setting:** a descriptive survey was conducted to determine challenges constraining sustainable household food security in the study area. The study took place within the Kasarani sub-county in Nairobi County, Kenya.

**Study population:** population comprised of 2060 urban farmers, 5 Agricultural Extension Officers (AEOs) and 5 Chiefs. The Chiefs and the AEOs were government officers familiar with the food security situation in the area under study. Inclusion of children was by consent of the parents this was between 13-15 years of age; children who gave oral consent and also got written consent for participation from their parents/legal guardian were included in the survey; only Saudi nationals were included in this study. Any medical condition that may lead to unreliable responses led to immediate exclusion as well those below 18 years were definitely excluded as well as anybody who failed to give/obtain consent to be part of this study. The study sample consisted of 384 urban farmers who were selected through a stratified random sampling method. The study sample also consisted of 5 Agricultural Extension Officers and 5 Administrative Chiefs chosen through the census sampling technique.

**Definition of variables:** the independent variables included socio-economic factors, gender dimensions, and prevailing challenges. The moderating variables were government policies and legal issues including laws, regulations, and policies. The dependent variable was household food security.

**Data collection:** data for the study was collected using an interview schedule for the Agricultural Extension Officers and Chiefs while a self-administered questionnaire with items that were both structured and unstructured. Content validity was established through expert judgments by university supervisors who offered their opinions after going through the designed data collection instruments and ascertaining them for data collection [7,8]. The researcher ascertained the internal consistency of the research instruments by making certain that the language used in the instruments was clear, simple, comprehensible, and free from any technical jargon. The internal consistency of the study tools was ascertained using the Cronbach Alpha Coefficient test and an Alpha Coefficient of reliability coefficients of 0.731 and 0.881 were obtained respectively for the two questionnaire variables was reported [9]. Data and methodological triangulation involving the usage of multiple methods such as documents, interviews, and previous studies helped the researcher to cross-verify the data and compare them with findings from different sources. Thus, the researcher was able to take in additional sources of information [8,9]. Conceptualization identified household food security as it presents a major cause of concern for urban dwellers economic planners and policy makers in many countries as a dependent variable. Socio-economic factors, gender dimensions, and prevailing challenges directly affected food security. For this to be governed and achieved then we need in place government policies and legal factors manifested in prevalence [5].

**Statistical analysis:** data were edited and coded in accordance with the application used and then put onto a matrix from which frequency tables were drawn and percentages derived. These were presented in a pictorial representation in the form of tables, graphs, and/or pie charts. Data for the study was analyzed using both descriptive and inferential statistics. Quantitative data was analyzed using both descriptive and inferential statistics. Descriptive statistics was used to describe and summarize the data in the form of graphs, tables, frequencies and percentages, while inferential statistics was used to help make inferences and draw conclusions. The Statistical Package for Social Sciences (SSPS) Version 22 was used to organize the data for interpretation.

**Ethical consideration:** an authorization to gather data was sought after from the Board of Postgraduate Studies of the University. Authorization was also sought from the Nairobi County Commissioner. Thereafter, the researcher booked appointments with the participants and explained the objectives of the study before the appropriate dates for conducting the study were determined. Before the questionnaire and the interview schedule were distributed to the participants, the researcher availed the Consent Form and requested them to sign it if they agreed to participate in the study. Each instrument was supplemented with a cover letter that introduced the researcher as well as thanking the respondent for taking part in the study. The information collected from the instruments formed the primary data for the study. Observation of ethical and professional considerations was done such as; seeking permission and clearance as well as strictly following procedures spelled out by Jomo Kenyatta University of Agriculture and Technology. In addition, Research permits will be sought from the Nairobi County Commissioner´s office and respective Chiefs and AEOs. Furthermore, Participants were guaranteed total confidentiality of the information provided and was used for research purposes.

## Results

**Participants:** the response rate from the respondents is shown in Table 1.

**Table 1:** response rates

Respondents	Number	Response rate
Urban farmers	328	85.42%
Agricultural extension officers	5	100%
Chiefs	5	100%

**Descriptive analysis:** all the sub-scales met the required level of internal consistency of reliability, with the Cronbach´s Alpha values ranging from a low of 0.721 (socio-economic factors questionnaire) to a high of 0.881 (challenges questionnaire).

**Figure 1:**
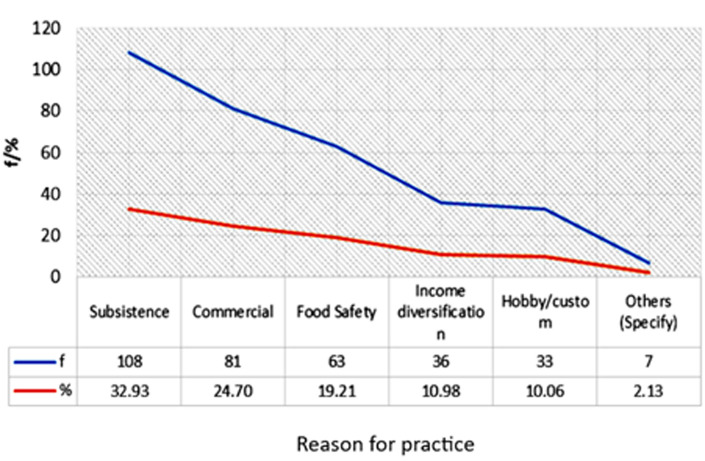
reasons for practicing urban farming

**Socio-demographic analysis:** the socio-demographic results are presented in Table 2 and Figure 1.

**Table 2:** socio-demographic characteristics

Characteristic	Result
Gender	Male- 65.55%;
	Female- 34.45%
Marital status	Married- 63.41%
	Divorced/separated- 23.78%
	Single or widowed- 12.81%
Level of education	Tertiary- 27.13%
	Secondary- 30.79%
	Primary- 28.05%
Occupation	Casual labourers- 41.46%
	Business people- 33.54%
	Formally employed- 25%
Experience in urban farming	6-15 yrs- 32.93%; 16-25 yrs- 28.35%; 26-35 yrs- 19.21%; <5 yrs - 17.38%; >35 yrs- 2.13%

**Socio-economic factors influencing household food security:** thirty-two point ninety-three (32.93), 24.7 and 19.21% of the respondents practiced urban farming for subsistence, commercial purposes, and food security, respectively. 10.98% practiced urban farming for income diversification while 12.19% did it either as a hobby/custom (10.06%) or some other unspecified purposes (2.13%) (Figure 1). 63.11% of respondents earned between 40- 80 USD 36.89% earned between 80 USD- 120 USD. The rest (14.02%) showed that they earned more than 120 USD (Table 3). The average mean rating was 3.63 (SD=0.86) in household food security before starting urban farming. The majority of the respondents were always worried that food would run out, at a rating of 3.85 (SD=0.98), with just a minority (12.10%) of the respondents holding a contrary opinion (Table 4). Fifty-four-point five percent of respondents felt that the food bought for the family did not last. Twenty-one point two zero percent of them strongly agreed that sometimes, children did not eat for whole days, reflecting a mean rating of 3.42 (SD=1.18). At a rating of 4.30 (SD=1.24), although more than 84.9% of the surveyed urban farmers were in agreement that they could not afford balanced meals prior to starting urban farming, a significant 24.20% of them did not agree on the statement. Likewise, although many (51.5%) of the urban farmers disclosed that adults in the house previously cut or skipped meals, 7.33% of them held a contrary view, reflecting a mean rating of 4.33 (SD=0.88) (Table 4).

**Table 3:** monthly income bracket from urban farming activities

Monthly income bracket from urban farming activities	F	%
Less than 40 usd	99	30.18
Between 40 usd- 80 usd	108	32.93
Between 80 usd-120 usd	75	22.87
More than 120 usd	46	14.02
Total	328	100.00
USD: United States dollar		

**Table 4:** household food security before starting urban farming

Household food security before starting urban farming	5	4	3	2	1	Mean	Standard deviation
I was always worried that food would run out	33.30	39.40	15.20	3.00	9.10	3.85	0.98
The food bought for the family didn’t last	54.60	30.30	9.10	3.00	3.00	4.30	1.24
I couldn’t afford balanced meals	33.30	24.20	8.20	12.10	12.10	3.55	1.12
My children were not eating enough	54.60	33.30	12.10	0.00	0.00	4.42	0.84
Adults in the house cut or skipped meals	51.50	36.40	9.10	0.00	3.00	4.33	0.88
You ate less than you felt you should	24.20	42.40	18.20	6.10	9.10	3.67	1.14
My family members were hungry because we didn’t have enough food to eat	39.40	27.30	9.10	15.20	9.10	3.73	1.18
Family members lost weight because of hunger	48.50	24.20	12.10	6.10	9.10	3.97	1.11
I cut the amount of children’s meals	39.40	27.30	15.20	18.20	0.00	3.88	1.21
My children were always hungry	51.50	27.30	12.10	3.00	6.10	4.15	0.96
My children did not eat for whole days	21.20	33.30	18.20	21.20	6.10	3.42	1.18
Mean average rating of household food security before starting urban farming						3.63	0.86

**Gender dimensions influencing household food security:** thirty-two point nine (32.09%) of female respondents and 24.35% of male respondents indicated they cultivate on a road reserve. 4.65% of the female respondents and 7.83% of the male respondents indicated on a railway reserve, 13.95% of the female respondents and 18.26% of the male respondents indicated on a public utility land, 9.77% of the female respondents and 13.91% of the male respondents indicated on my own land, 14.42% of the female respondents and 15.65% of the male respondents indicated on rented land, 20.93% of the female respondents and 18.26% of the male respondents indicated along the river bank while 4.19% of the female respondents and 1.74% of the male respondents indicated that their urban farms were located on other unspecified locations (Table 5). The cultivating lands were either inherited, bought, allocated, rented, or acquired in other unspecified means with different percentages for both males and females Furthermore, the crops grown were beans, English potatoes, carrots, beetroots, maize, pumpkins, cabbages, spinach, sweet potatoes, kales, onion, lettuce, and other unspecified crops (Table 5). The livestock kept were poultry, sheep, pigs, cattle, and other unspecified livestock. Males and females perform different functions in urban firms such as bush clearing, raising beds, and transplanting, amongst others (Table 5).

**Table 5:** gender dimensions Influencing household food security

	Female (n=215)		Male (n=113)	
	F	%	f	%
**Location of urban farms**				
On a road reserve	69	32.09	28	24.35
On a railway reserve	10	4.65	9	7.83
On a public utility land	30	13.95	21	18.26
On my own land	21	9.77	16	13.91
On a rented land	31	14.42	18	15.65
Along the river bank	45	20.93	21	18.26
Others (specify)	9	4.19	2	1.74
**How urban farms were acquired**				
Inherited	96	44.65	38	33.04
Bought	25	11.63	25	21.74
Allocated	43	20.00	24	20.87
Rented	31	14.42	18	15.65
Others (specify)	20	9.30	10	8.70
**Crops grown in urban farms**				
Beans	53	24.65	26	23.01
English potatoes	62	28.84	36	31.86
Carrots	34	15.81	12	10.62
Beetroots	11	5.12	9	7.96
Maize	56	26.05	38	33.63
Pumpkins	29	13.49	24	21.24
Cabbages	51	23.72	34	30.09
Spinach	49	22.79	22	19.47
Sweet potatoes	23	10.70	41	36.28
Kales	31	14.42	31	27.43
Onion	29	13.49	27	23.89
Lettuce	43	20.00	30	26.55
**Gender dimensions influencing household food security; livestock kept in urban farms**				
Poultry				
Sheep	185	86.05	89	78.76
Pigs	90	41.86	73	64.60
Cattle	13	6.05	57	50.44
Other (specify)	8	3.72	7	6.19
	5	2.33	6	5.31
**Tasks associated to respondents’ gender**				
Clearing the bush				
Raising beds	22	10.23	97	85.84
Planting and transplanting	136	63.26	105	92.92
Weeding	157	73.02	91	80.53
Fertilising	169	78.60	89	78.76
Spraying	178	82.79	73	64.60
Manual watering	59	27.44	90	79.65
Harvesting	68	31.63	35	30.97
	208	96.74	107	94.69

**Challenges constraining sustainable household food security:** the exploratory data analysis revealed that urban farmers were mostly constrained by flooding (MN=3.66) followed by lack of rain (MN=3.52), pests/diseases (MN=3.49), poor yields (MN=3.28) and soil erosion (3.23) (Table 6). The analysis revealed that most respondents considered city authorities harassment (MN=0.79), lack of land security (MN=0.78 and lack of access to land (0.76) the greatest challenge to sustainable household food security (Table 7).

**Table 6:** natural challenges to urban farming and sustainable household food security

Challenges constraining sustainable household food security	1	2	3	4	5	Mean	Standard deviation
Lack of rain	6.40	4.27	8.54	29.27	36.89	3.52	2.84
Flooding	9.15	4.88	10.67	21.34	34.15	3.66	2.92
Soil erosion	2.74	4.57	19.82	23.78	29.88	3.23	2.14
Pests/diseases	28.96	27.13	15.24	10.37	5.49	3.49	1.84
Poor yields	23.48	33.54	7.32	13.11	4.57	3.28	2.15
Mean average rating on natural challenges to urban farming						3.44	2.38

**Table 7:** challenge to sustainable household food security

Challenges with urban farming	1	2	3	4	5	MN	SD
Lack of access to land	32.32	28.66	8.23	11.59	13.72	0.76	2.37
Lack of land security	34.76	29.57	14.02	10.67	7.93	0.78	2.63
City authorities’ harassment	32.32	4.57	19.82	17.68	23.78	0.79	2.21
Transportation	28.96	27.13	15.24	10.37	5.49	0.70	2.25
Theft of crops	23.48	33.54	7.32	13.11	4.57	0.66	2.63
Lack of assisting labour	30.37	24.70	12.93	12.68	11.10	0.73	2.42
Lack of access to food for livestock	32.32	28.66	8.23	11.59	13.72	0.73	1.82
Plot used as toilet	34.76	29.57	14.02	10.67	7.93	0.72	1.66
Mean average rating to challenge to sustainable household food security						0.73	2.25

## Discussion

The study aimed to determine the challenges constraining sustainable household food security in Kasarani sub-county in Nairobi Kenya. It was established that flooding, lack of rain, pests/diseases, poor yields, and soil erosion were the major factors that affected urban farming. Other factors included harassment from city authorities, lack of land security, and lack of access to land. The 98% response rate is important to the credibility of the research results as it bears on the representativeness of the selected sample and a response rate of 80% and above is deemed sufficient in making generalizations about the entire population [8]. The response rate of a study helps the researcher to gauge the potential for non-response bias [9]. The high response rates in the current study infer that the views collected from the respondents reflected elements of the population with breadth and depth and is therefore representative and reliable [10]. Cronbach´s Alpha Coefficient analysis was used to measure the internal consistency of the instruments because it is the most consistent test of inter-item consistency reliability for Likert-scaled or rating-scaled questionnaires. Internal consistency is the degree to which an instrument is error-free, reliable, and consistent across time and across the various items in the scale [9]. The reliability for multi-item opinion items was computed separately for the entire sub-scales in the urban farmers questionnaires and the coefficient alpha of these variables was reported.

The findings of the Cronbach´s Alpha test were in line with the recommendation that a coefficient of 0.60 is of adequate reliability while a coefficient of 0.70 and above indicates that the instrument has a high inter-item consistency reliability standard [7-9]. Therefore, the questionnaires were suitable for data collection because they adequately measured the constructs for which they were intended. Based on socio-demographic results, the younger cohort of school leaders embrace change and exhibit great skills at marketing their ideas [11-13]. They are also more optimistic about their change proposals and therefore relatively more excited about accomplishing academic standards in their respective schools [14]. Education may influence income as it plays a key role in the choice of jobs [15,16]. Studies also assert that urban dwellers with low levels of education are usually more likely to acquire more gardens to boost their income levels and provide food for subsistence in their homes [17,18]. This is unlike their counterparts who have higher levels of education and are gainfully employed with sufficient incomes to sustain their families. They may not therefore need many gardens to produce crops for food and sale. Another argument in a scholarly study shows urban farmers, their spatial distribution and characteristics, cultivation practices, crop types, consumption patterns, and crop produce disposal [19-21].

The insinuation here is that urban farming has progressively advanced into a major source of employment for urban dwellers who adopt it after failing to secure formal employment, either as farmers or fresh vegetable sellers in urban markets. For example, a recent case study carried out in Nairobi and Kisumu disclosed that urban livestock keeping contributed to food security, income, employment generation, savings a system of insurance, and social status [17,18,22]. The study concluded that though still undervalued and resisted by county government public officials, urban farming has gained a widespread and long-established occupational activity in urban centers.It can be deduced from the results that the urban farmers who participated in this study had served long enough and were therefore familiar with insights into the different practices and provided the experience and alternative options. Personal characteristics such as experience in urban farming and access to resources may positively and negatively influence perceived behavioral control and influence urban food producers´ intention to continue farming in urban settings [23-25]. The results on reasons for participating in urban farming agree with the findings of a scholarly study which concluded that people have unlike motives for partaking in urban gardening including curiosity towards growing food and seeing how much can be produced (in Finland) [26]. Learning about cultivation, raising awareness on urban gardening and hopefully contributing to an increase, growing plants that are not sold in the shops, experimenting, pioneering, rebelliousness, influencing the society, and shaping the cityscape [12].

The average mean rating of 3.63 (SD=0.86) in household food security before starting urban farming showed a gap in household food security before respondents started urban farming, given that it has only moderate level of food security. This implies that urban farming has made it possible, especially for poor households, to improve food security by providing healthy and plentiful substitutes for purchased food. This was demonstrated by the large portion of respondents who rated most of the items as above average [27-32]. Poor households are more food secure and have better nutritional status than non-farming households of similar socio-economic status when they produce their own food. Furthermore, production for domestic ingestion and sale enable households to generate income and reduce monthly expenses on food. As a result, the households are left with more funds for other basic household needs including health, housing, education and clothing [27,29,30,32].

Females are seen to be more than males had a stronger determination to use coping mechanisms that enable them to harness resources and avail food for their families [33]. The study recommended the need to empower women in Urban Farming to become better food providers and achieve better food security for their households. However, this notion is discredited in a study that suggested that in the last few years, there have been more male farmers engaging in UPA activities [33-36]. Following acceptance and apparent protection by the Nairobi Urban Agriculture Promotion and Protection Act of 2014 therefore, provides food security, income, and employment to many unemployed men to feed their families [37-39]. According to gender inequality is usually associated with food insecurity and worldwide, close to 60% of the chronically hungry people are women and girls. This notwithstanding that they are the ones that are responsible for preparing meals for the families and taking care of the children [11,13,30]. Women, many times, spend all or a substantial amount of their income either on feeding or paying for their children´s needs [40,41]. Women-headed households are habitually more susceptible to poverty and hunger than households headed by a man because women oftentimes lack the ability to command labor within and outside the household [42]. Some analysts even suggest that yield would increase by 20% - 30% if women were to have similar access to productive resources as men. This would boost the total agricultural output in developing countries by a significant 2.5% to 4%.

Based on the results of challenges affecting sustainable household security, they seem to oppose the findings of a study by Sage that found that limited lateral space, high land values, contaminated soils, theft and vandalism, and pavement encroachment were among the commonest challenges to every city everywhere [29]. Other than these observations show that loss and damage of crops from birds and rodents, high costs (water, infrastructure, permits, housing, etc.), and lack of experienced skilled labor and management [30]. On a similar note, long-term natural resource constraints, specifically water, land and forests, soils, biodiversity, and fisheries are named as some natural factors that are making it more difficult to increase productivity, especially for key food crops [1,17]. A study by the National Research Council (2012) names a lack of investment, slowing growth in yields, and declining availability of funds for research and development as some of the major challenges to sustainable household food security in the 21^st^ Century [31].

Other challenges mentioned include low significance accorded to agriculture by many governments, insufficient international financial support to agriculture and agricultural research, institutional and infrastructure barriers to action by the private sector, including smallholders together with continued natural resource degradation and location-specific challenges [32]. The study had its strengths and weaknesses. Due to time limitations and a large sample size, the researcher was unable to interview all personnel involved in urban farming and urban household food security in the sub-county. The researcher was unable to carry out a cross-sectional study of similar initiatives outside Kasarani sub-county, Kenya due to financial and time constraints. For this reason, the results may contain generalizations of the findings of the study. Third, the researcher was unable to get information on critical areas, which respondents may consider personal or confidential. The researcher tackled the restrictions by guaranteeing respondents of the confidential nature of the study and assured them that the study results were intended for academic purposes only. The investigator also set aside extra time for respondents coming from areas with sampling errors in order to accommodate any possibilities for failure.

## Conclusion

The study established the challenges to household food security within Kasarani sub-county in Nairobi, Kenya. Urban farming helps urban communities in various social and economic ways that stimulate the local economy. Sufficing that the food products are meant for domestic consumption, urban farming serves as an effective means of meeting the food security the food security needs of urban dwellers. In the case of Kasarani sub-county, urban farming also offers farmers the means to avail better nutrition, alleviate poverty, create employment, reduce pressure on finite farmland and conserve the environment. The study recommends adherence to weather forecasting to reduce on the effects of flooding, adoption of measures to prevent soil erosion and usage of pesticides whenever applicable. A policy and institutional framework for the sector need to be established to enable urban farmers to mutually benefit from urban farming.

### What is known about this topic

- Urban farming is practiced very much;
- Government policy has influenced the urban farming greatly.

### What this study adds

- Contribute to policy formulation;
- Knowledge, attitude, innovation, adoption of technology.
